# Plasma-Enabled Carbon Nanostructures for Early Diagnosis of Neurodegenerative Diseases

**DOI:** 10.3390/ma7074896

**Published:** 2014-06-25

**Authors:** Shafique Pineda, Zhao Jun Han, Kostya (Ken) Ostrikov

**Affiliations:** 1 Plasma Nanoscience Centre Australia (PNCA), CSIRO Materials Science and Engineering, P.O. Box 218, Lindfield, NSW 2070, Australia; E-Mails: shafique.pineda@csiro.au (S.P.); kostya.ostrikov@csiro.au (K.K.O.); 2 Plasma Nanoscience@Complex Systems, School of Physics, the University of Sydney, Sydney, NSW 2006, Australia; 3 School of Chemistry, Physics, and Mechanical Engineering, Queensland University of Technology, Brisbane, QLD 4000, Australia

**Keywords:** low-temperature plasmas, carbon nanostructures, plasma nanoscience, graphene, nanotubes, nanosheets, nanowalls, biosensor, neurodegenerative diseases

## Abstract

Carbon nanostructures (CNs) are amongst the most promising biorecognition nanomaterials due to their unprecedented optical, electrical and structural properties. As such, CNs may be harnessed to tackle the detrimental public health and socio-economic adversities associated with neurodegenerative diseases (NDs). In particular, CNs may be tailored for a specific determination of biomarkers indicative of NDs. However, the realization of such a biosensor represents a significant technological challenge in the uniform fabrication of CNs with outstanding qualities in order to facilitate a highly-sensitive detection of biomarkers suspended in complex biological environments. Notably, the versatility of plasma-based techniques for the synthesis and surface modification of CNs may be embraced to optimize the biorecognition performance and capabilities. This review surveys the recent advances in CN-based biosensors, and highlights the benefits of plasma-processing techniques to enable, enhance, and tailor the performance and optimize the fabrication of CNs, towards the construction of biosensors with unparalleled performance for the early diagnosis of NDs, via a plethora of energy-efficient, environmentally-benign, and inexpensive approaches.

## 1. Introduction

Neurodegenerative diseases (NDs) such as Parkinson’s disease (PD) and Alzheimer’s disease (AD) are becoming more prevalent with the ageing population. At present, over 40 million individuals worldwide are affected by AD or PD [1,2]. More critically, this amassing population of sufferers may see a three-fold increase by 2050 [3,4]. In addition to human suffering, this represents not only a significant loss in productivity, but also a huge burden on the heath care system and the economy [5]. Whilst a cure is not yet available, the symptoms associated with ND may be more effectively managed with early intervention therapies and lifestyle adjustments if they are diagnosed *as early as possible*.

Unfortunately, NDs are usually first diagnosed symptomatically, once the disease has progressed to the point where it is noticeable. The identification of clinical markers (*i.e.*, Lewy bodies in the PD case [6] and Lewy-body dementia or plaques in the AD case [7,8]) is typically only possible post-mortem. Biomarkers, on the other hand, are good candidates for the early detection of ND. Early detection will enable medical practitioners to implement treatment strategies such as brain exercises and physical fitness regimes, and identify sufferers who may respond to drugs (e.g., for AD [9,10,11], and PD [12,13,14]) designed to slow the progression of the disease. This points to the critical importance of developing practical advanced sensors capable of detecting low concentrations of ND-related biomarkers (including, but not limited to, beta-Amyloid (Aβ) [15,16], alpha-Synuclein [17,18], mtDNA [19], and phospholipids [20]), and distinguish between their various mutated and misfolded forms (e.g., monomers, protofibrils, oligomers, and fibrils) in complex mixtures such as blood and cerebrospinal fluid (CSF), for a presymptomatic diagnosis and progressive monitoring of AD and PD.

### 1.1. Biosensors

Fundamentally, a biosensor is a device comprised of two elements—a biomolecular receptor capable of specific reaction with the target analyte, and a transducer which processes the biorecognition event into a measurable signal. Indeed, a diverse spectrum of electrochemical [21,22,23,24,25,26] and optoelectrical [27,28,29] biosensors has been recently developed for the detection of biomarkers indicative of AD or PD. However, these biosensors fall short in addressing one or more key criteria of device performance and fabrication. In particular, this includes a high sensitivity and specificity in physiochemical environments (*i.e.*, blood or CSF), a rapid sensor response and regeneration, and an inexpensive device fabrication. Consequently, future innovations in the design of novel advanced materials towards the optimization of biosensors for ND biomarkers remain highly warranted.

### 1.2. Advanced Materials: Carbon Nanostructures

Notably, carbon nanostructures (CNs) (e.g., nanotubes, nanosheets, and nanowalls) inherit a multitude of characteristics that enable their versatility to simultaneously play the role of a specific receptor and a sensitive transducer. For instance, the mechanical robustness, high electrical conductivity, superior optical properties, and ease of functionality of CNs, have been harnessed to realize a plethora of hierarchical biorecognition nanocomposites (see Section 2.1, Section 2.2, Section 3.1, Section 3.2, and Section 4.1 below) which may be tailored for the determination of AD or PD biomarkers. Correspondingly, this highlights the necessity for a deterministic, resource-efficient, and practical synthesis for these hybrid nanostructures.

### 1.3. From Fabrication to Performance: The Plasma Advantage

Conventional approaches for the synthesis of carbon-based biorecognition structures have involved techniques such as lithography, wet chemical processing, and chemical vapor deposition (CVD). However, not only are these methods time consuming and energy-inefficient, but also the harsh environments involved have been responsible for inducing substrate damage, unwanted impurities, and non-uniformity in yield. Consequently, this leads to a poor reproducibility in device fabrication and a compromised biosensing performance. On the other hand, plasma-based techniques [30,31,32,33] for fabrication and surface treatment may be implemented to enable, enhance, and tailor the device performance and optimize the fabrication processes of CNs towards the construction of biosensors with superior properties, via a plethora of resource-efficient, eco-friendly, and inexpensive approaches (see Section 2.3, Section 3.3, and Section 4.2 below).

## 2. Graphene

Graphene, a single-layer two-dimensional (2D) honeycomb lattice of *sp*^2^-hybridized carbon atoms [34,35,36], inherits an exceptional tensile strength [37], large specific surface area [38,39,40], ease of functionality [41,42], tunable band gap [43,44,45], low electrical noise [46,47], ballistic charge transport [48,49], and high charge carrier concentrations at room temperature [46,50]. Consequently, a wide variety of graphene-based electrical (Section 2.1) and optical (Section 2.2) biosensors have been realized through its unique physicochemical properties.

### 2.1. Graphene-Based Electrical Biosensors

Graphene-based field effect transistors (G-FETs) have been essential towards the developments for a label-free and real-time determination of biological complexes. In G-FETs, the surface of graphene serves as the interfacial element for biorecognition and transduction. Typically, target analytes are selectively detected by a specific binding with complementary biomolecules functionalized on the surface of graphene. In particular, a charged molecule (e.g., protein, DNA) adsorbed on the surface of graphene induces a perturbation in the local charge density, leading to a change in the channel conductance.

In G-FET DNA biosensors, selectivity is enabled through the hybridization between complementary DNAs. For instance, single-stranded (ss) DNAs have been tethered on the surface of chemically modified graphene through linker molecules for a specific detection of the complementary DNA [51,52,53,54]. Notably, the first G-FET DNA biosensor was established by Mohanty *et al.* [51], where graphene oxide (GO) sheets were prepared by a modified Hummers method [55,56,57] and backgated with silica. The functional groups on GO were utilized to immobilize single-stranded fluorescent DNA probes for a selective detection of complementary target DNAs through successive base-pairing hybridization/dehybridization interactions and restorations in GO conductivity. Subsequently, innovations in DNA G-FETs including electrolyte-gated setups [58,59], CVD-prepared graphene for low-noise performance [60], and an increased loading of probe DNAs through nanoparticle (NP) functionalized graphene-based substrates [58,59], have driven improvements in the detection limit of DNAs to the nM and pM concentrations. Most recently, Cai *et al.* [61] have designed a reduced graphene oxide (RGO)-FET DNA biosensor capable of selectively identifying single-based mismatched DNAs and noncomplementary DNAs, with a resolution of 100 fM, which is one order of magnitude lower than previously reported G-FET DNA biosensors. In this biosensor, peptide nucleic acid (PNA) (instead of ssDNA) was employed as the capture probe, and DNA recognition was achieved through PNA-DNA hybridization on the RGO surface. Above all, these G-FETs with the capability of sequence-specific DNA recognition may be tailored for a label-free and real-time determination of DNA mutations indicative of AD or PD.

**Figure 1:**
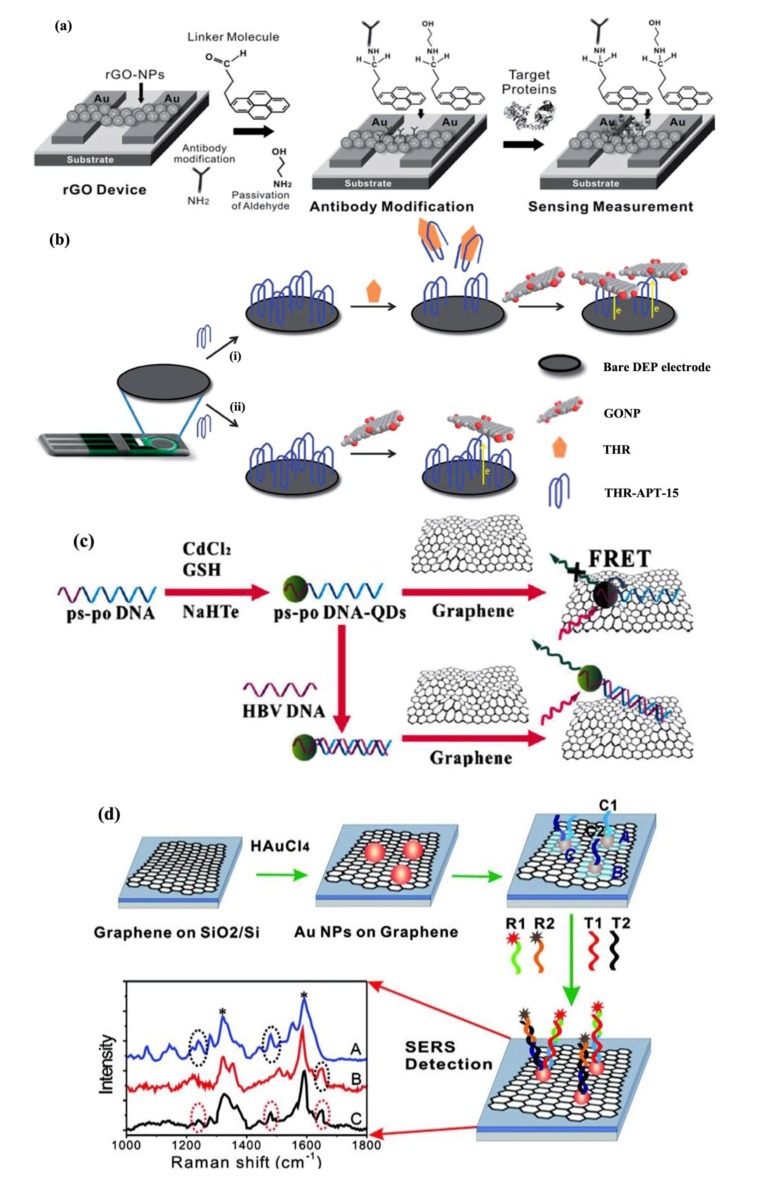
Graphene-based field effect transistors (FET), electrochemical, fluorescent resonance energy transfer (FRET), and surface enhanced Raman scattering (SERS)-active biosensing platforms. (**a**) A reduced graphene oxide (RGO)-encapsulated nanoparticle (NP) array for a highly sensitive, selective and label-free detection of breast cancer biomarkers. Reproduced from [70]; (**b**) Graphene oxide nanoplatelets (GONPs) utilized as electroactive labels for the detection of thrombin (THR). Reproduced from [91]; (**c**) Schematic for the preparation of DNA-CdTe quantum dots (QDs) for a FRET assay of DNAs. Reproduced from [92]; (**d**) Preparation of a multiplexed SERS-active substrate based on AuNP-decorated DNAs. Reproduced from [93].

A similar biorecognition protocol has been developed for proteins (*i.e.*, immunoglobulins), where selectivity was enabled through antibody-antigen [62,63,64,65] or protein-aptamer [66,67,68,69] binding at the graphene interface. Moreover, the probe biomolecules (e.g., antibodies, aptamers, ssDNAs) may be labeled on the surface of chemically modified graphene via (i) chemical linkers [65,67,70]; or (ii) conjugation with NPs adsorbed on its surface [63,64,70]. The chemical-linker-based approach has been adopted by Kurkina *et al.* [65] to realize a RGO-FET immunosensor for Aβ peptides. The surface of RGO was initially functionalized with *Staphylococcus aureus* protein A (SpA) through carbodiimide coupling [71], and subsequently, anti-Aβ-antibodies were immobilized on SpA-RGO through the specificity of SpA with the *Fc* antibody fragments [72]. This immunosensor reported a detection limit of 1 fM Aβ—an order of magnitude improvement compared to commercial ELISA assays for Aβ quantification in biological fluids [73]. In the second approach based on conjugated NPs, not only do NPs increase the load of probe biomolecules, it also enables an outward orientation of binding sites (*i.e.*, antibody Fab region), and hence optimizes the device sensitivity by maximizing the number of available capture sites for the target proteins. For instance, Mao *et al.* [63,64] and Myung *et al.* [70] have established a highly-sensitive and selective G-FET protein biosensor, based on RGO decorated with AuNP-antibody conjugates for the determination of complementary antigens. The biorecognition substrate was also prepared by thermal reduction from GO [63], whereby a detection limit in the order of ng/mL was deduced, and the specificity in antibody-antigen binding mechanism was demonstrated for the immobilization of non-complementary antigens including immunoglobulin-G (Ig-G), immunoglobulin-M (Ig-M) and horseradish peroxide (HRP). In Figure 1a the fabrication of a similar NP composite-based G-FET immunosensor [70] is illustrated, where reduced graphene oxide (RGO)-encapsulated nanoparticle (NP) arrays were implemented for a highly sensitive, selective and label-free detection of breast cancer biomarkers. Moreover, the limit of protein detection may be enhanced by reducing the effects of Debye screening on the immobilized target proteins. In particular, this has been demonstrated by Kim *et al.* [62,68], where aptamers (short oligonucleotides) were utilized as probe biomolecules to achieve detection limits in the femto- and atto-molar ranges.

On the other hand, graphene-based electrochemical (GEC) biosensors rely on redox reactions between the probe and target biomolecules for transduction. In comparison with G-FETs, GEC biosensors are attractive as they present a simpler and hence less expensive electrode fabrication. Consequently, a diverse spectrum of highly-selective and sensitive GEC biosensors have been developed for the determination of disease-specific protein biomarkers [74,75,76,77], in conjunction with nucleic acids (*i.e.*, DNA bases) and DNA sequences [78,79,80,81,82,83,84,85,86,87,88,89,90], which may be tailored for the realization of single nucleotide polymorphism (SNP) assays for DNA mutations correlated with the development of AD or PD.

The properties of graphene, *namely*, its diverse functionalities, two-dimensional structure, and high electrical conductivity, have been crucial towards the enhancement in selectivity and sensitivity of electrochemical biosensors. In particular, graphene is typically integrated as an electrode coating in GEC biosensors as its surface may be functionalized with chemical linkers [75,76,77,94] or nanocomposites (e.g., nanoparticles, nanorods) [87,89,95], to facilitate the adsorption or immobilization of probe biomolecules (e.g., antibodies, ssDNAs) for a highly-sensitive and specific assay of the target analytes. For instance, a label-free GEC immunosensor with a resolution of 0.012 U/mL for the cancer biomarker, carbohydrate antigen 15-3 (CA 15-3), was reported [75]. A glassy carbon electrode was coated with nitrogen-doped graphene sheets for the immobilization of CA 15-3 specific antibodies through its *Fc* site. The modified electrode was subsequently immersed in BSA to prevent non-specific binding, before the target antigens were introduced. While metal nanoparticles have been commonly employed as electroactive labels in electrochemical sandwich assays [96,97], a novel aptasensor which exploits the inherent electroactivity of graphene oxide nanoplatelets (GONPs) for labeling was recently developed (Figure 1b) [91]. In this assay, the basis of detection lies in the ability of GO to be electrochemically reduced to provide a well-defined reduction “wave”, in which a single GONP (dimension 50 × 50 nm) induces a reduction signal by accepting electrons from the modified electrode surface. By using GONPs as an inherently electroactive label, thrombin detection was demonstrated in the concentration range of 3 pM to 0.3 µM, with good selectivity of the aptamer towards interferences by bovine serum albumin (BSA), IgG and avidin. Most importantly, these GEC biorecognition platforms may be adapted to the determination of protein biomarkers and DNA mutations indicative of AD or PD.

### 2.2. Graphene-Based Optical Biosensors

Optical biosensors have predominantly capitalized on the surface plasmonic [98,99] and dye-quenching properties [100,101,102] of graphene to design a diverse range of highly-sensitive, very specific, extremely fast and low-noise biosensors for proteins and DNAs. The mechanisms involved include fluorescent resonance energy transfer (FRET) [103,104,105,106,107], surface plasmon resonance (SPR) [108,109,110,111], surface enhanced Raman scattering (SERS) [93,112,113], and several other protocols.

By exploiting the photoluminescence of GO and quenching ability of gold nanoparticles (AuNPs) for organic fluorophores, a series of FRET-based biosensors of GO arrays were realized towards aptasensing [105] and immunosensing [106] of proteins, and for the detection of sequence-specific DNA hybridization [107,114]. Correspondingly, probe biomolecules including aptamers [105], antibodies [106], ssDNAs and molecular beacons (MBs) [115,116] have been utilized in these selective FRET assays of proteins and nucleic acids (e.g., DNA bases and sequences). Generally, the capture of a target biomolecule is verified by the fluorescence quenching of GO by FRET between the fluorescent (*i.e.*, NP)-tagged target and GO sheets. In Figure 1c, a typical FRET assay is illustrated for DNA-CdTe quantum dot (QD) conjugates with graphene [89]. In the case of Liu *et al.* [107], probe DNAs linked to the surface of GO by carbodiimide chemistry were hybridized with AuNPs labeled with the complementary target DNA strands, which correspondingly led to a decrease in fluorescence emission intensity from the GO arrays. Based on a similar principle of FRET, Jung *et al.* [106] have fabricated an alternate biorecognition assay for immunosensing. This biosensor was realized through the synthesis of AuNP-antibody (Ab) conjugates (Ab-AuNP-DNA complexes) via ssDNA linkers. In particular, DNA linkers were utilized to provide a control of distance between Ab and AuNPs, such that AuNPs could quench the fluorescence while bound to the GO surface. The high affinity of amino functional groups of DNA nucleotides for AuNPs led to an enhancement in the quenching efficiency of GO. Consequently, target biomolecules on the GO surface were identified through a specific binding with Ab-dsDNA-AuNP complexes, which resulted in a reduction of fluorescence emission by the GO arrays. Furthermore, while fluorescent tags such as quantum dots [104,117] and NPs have been commonly employed in FRET-based assays, a novel protocol for sequence-specific DNA sensing was recently developed [103], which utilizes the fluorescence from aggregation-induced emission (AIE) molecules. In particular, 9,10-distyrylanthracene with two ammonium groups (DSAI) were used in a novel AIE probe, and the fluorescent aptasensor based on DSAI and GO was developed for selective and sensitive detection of targeted DNA and thrombin proteins.

On the other hand, SPR biosensors utilize the propagation of surface plasmon polariton (SPP) waves to probe the interactions between biomolecules and the sensing surface. In particular, graphene has been employed as a functional coating to enhance the SPR signal and the immobilization of probe biomolecules. For instance, Wu *et al.* [111] have achieved a superior performance in biomolecular recognition for graphene-on-gold compared to gold-only platforms in SPR-based biosensors. As the graphene coating improves the propagation of surface plasmon polaritons, it consequently enhances the device sensitivity to changes in its refractive index. Additionally, the detection of biomolecules with carbon-based ring structures (e.g., ssDNA) was discovered to be well supported by graphene-on-Au SPR biosensors. This stems from an increased bioaffinity to the surface of graphene through π-stacking of *sp*^2^ orbitals, and consequently enhances the device sensitivity. On the detection of proteins, Zhang *et al.* [109,110] have established an SPR biosensor composed of GO decorated with gold nanostrucutre-antibody conjugates for a specific measurement of the antigen content. The large specific surface area and abundant oxygen-containing functional groups of GO had facilitated the immobilization of gold nanorod (AuNR)–antibody conjugates on its surface [110]. Meanwhile, AuNRs anchored to the GO surface function as SPR signal enhancers which greatly improved sensitivities in the determination of transferrin, as evident by the limit of detection which was reported to be 32 times lower than that obtained with continuous Au films. Moreover, Wang *et al.* [108] have demonstrated a label-free and regenerative SPR biosensor for α-thrombin, composed of noncovalently adsorbed α-thombin-specific aptamers to the surface of reduced GO (RGO) coated Au films. The detection mechanism involves a detachment of α-thrombin-specific aptamers from the SPR biorecognition surface upon binding with α-thrombin, and leads to a noticeable change in the SPR resonant angle within a detection limit of 0.05 nM.

Indeed, as the surface of graphene may be decorated with NPs to amplify the SPP waves, this facilitates its applicability to SERS-based substrates for single molecule detection [93,96,112,113,118]. For instance, Liu *et al.* [119] have designed an AgNP functionalized GO for an ultrasensitive detection of aromatic molecules with various charges including, positively charged crystal violet (CV), negatively charged amaranth, and neutral phosphorus triphenyl (PPh3). This may be adapted for the determination of charged molecules including proteins and DNAs (phosphate backbone). Furthermore, Lu *et al.* [113] have established a SERS substrate based on an RGO film decorated with silver (Ag) NPs for the sensing of aromatic molecules. In comparison with graphene-only substrates, this AgNP-RGO composite shows a superior sensitivity, and a detection limit in the order of nM. Moreover, this may be harnessed to monitor the levels of aromatic residues namely, Amyloid-related proteins and short peptide fragments, which precede the formation of Amyloid fibrils in AD. An analogous setup for DNAs was tailored by He *et al.* [93] (Figure 1d), in which a multiplexed and sequence-specific detection was achieved. In particular, this involved a SERS-active substrate based on AuNP-decorated CVD-growth graphene. Due to the combination of AuNPs and graphene, the Raman signals of dye were electrochemically enhanced [120,121] by this novel substrate. With the AuNPs, DNA capture probes were assembled on the surface of graphene films. Consequently, it was reported that two different DNA targets sharing a substrate could be simultaneously determined (down to 10 pM) with a single light source.

### 2.3. Plasma-Processing: Graphene

**Figure 2:**
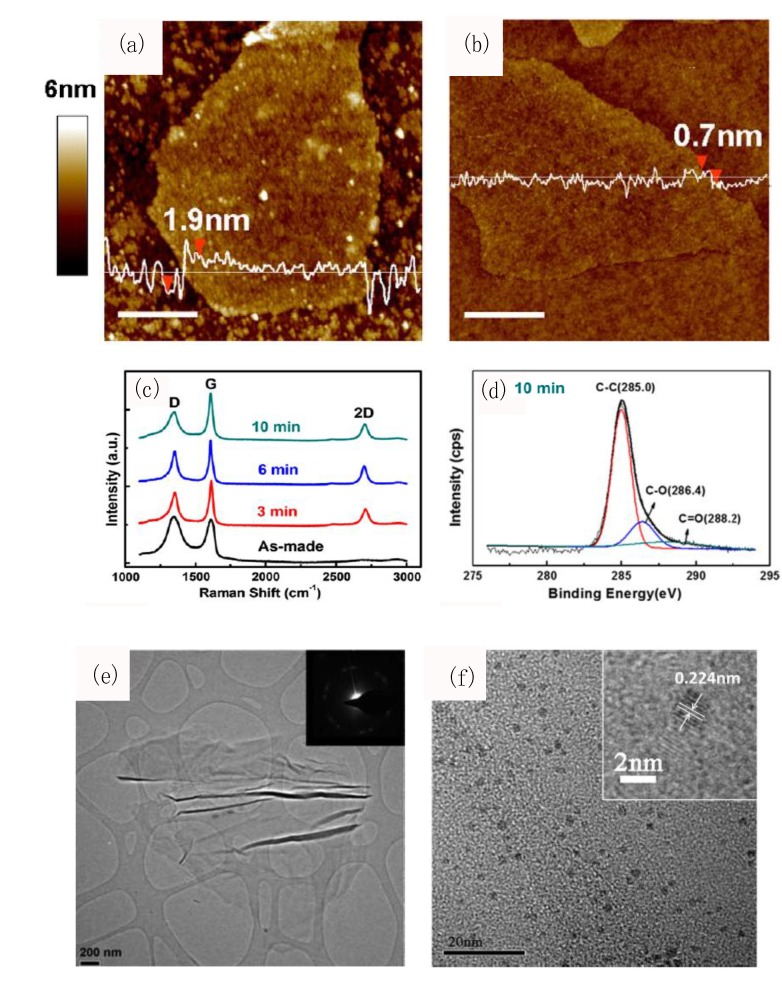
Structures and morphologies of graphene oxide (GO) under plasma-assisted reduction and decoration with nanoparticles (NPs). Atomic force microscopy (AFM) characterization of (**a**) GO and (**b**) reduced graphene oxide (RGO) sheets after 10 min of H_2_ plasma treatment at 525 °C; (**c**) The Raman spectra showing the quality and impact of plasma-assisted reduction of GO (*i.e.*, “as-made”) to RGO; (**d**) The C1 XPS spectra of GO after 10 min of plasma treatment. Reproduced from [123]; (**e**) Transmission electron microscopy (TEM) image of nitrogen-doped graphene. Inset shows the selected-area electron diffraction (SAED). Reproduced from [136]; (**f**) High-resolution TEM image of GO nanosheets uniformly decorated with palladium (Pd)-NPs via a low temperature plasma-assisted fabrication. Reproduced from [137].

The importance of plasma-treatment in graphene-based biosensors is three-fold, *namely*, to assist fabrication, enhance sensitivities, and enable the tailoring of functionalities, via rapid, scalable, versatile, and contaminant-free procedures (Figure 2). For instance, the synthesis of graphene via (H_2_/CH_4_) plasma-enabled chemical vapor deposition (PECVD) on Cu [122] or atmospheric pressured (CH_4_/H_2_/Ar) PECVD on SiO_2_ [123,124] present an energy efficient, high-quality yield and low temperature (~600 °C) alternative to the conventional methods such as thermal CVD (900–1000 °C) [125], thermal decomposition of SiC wafer [126], mechanical exfoliation of bi/tri-layer graphene [127,128], thermal annealing [129,130,131], and chemical [129,132,133] and photocatalytic [134,135] reduction from graphene oxide. On the other hand, surface functional groups of nanostructures created in a plasma environment are dependent on the active species and the specific plasma conditions. For example, high-pressure (e.g., at atmospheric pressure) plasmas features an environment with low ion collision frequencies, hence reducing damages on nanostructures induced by the highly energetic ions. By avoiding complex vacuum technologies, these high-pressure plasmas are also suitable for direct in-line surface modifications. In contrast, low-pressure plasmas are advantageous in providing a high uniform and deep treatment to the nanostructures, due to the uniform processing environment with less ion collisions under the high vacuum.

Notably, the restoration of GO may be most efficiently achieved via plasma-treatment [123], as evidenced by the superior electrical conductivity of RGO and its structural characterization illustrated in Figure 2a–d. Flakes of GO (Figure 2a) were treated by H_2_ plasma (10 mins at 525 °C), for the production of highly uniform reduced graphene oxide (RGO) sheets (Figure 2b). Correspondingly, the Raman spectra in Figure 2c demonstrates the quality and impact of plasma-assisted reduction of GO (*i.e.*, “as-made”) to RGO. The plasma-assisted restoration of GO results in a decrease in *I_D_/I_G_* (D/G peak intensity) ratio from 1.03 (“as-made”) to 0.53 after 10 min of plasma exposure. As *I_D_/I_G_* is proportional to the average size of the *sp*^2^ carbon domains, this decrease in *I_D_/I_G_* is attributed to the removal of defects and the conversion of *sp*^3^ to *sp*^2^ carbons. In Figure 2d, the C1 XPS spectra of GO after 10 min of plasma treatment further highlights the superior quality of graphene obtained *via* plasma-assisted reduction. In particular, a dominant C-C peak corresponds to the removal of most oxygen functional groups. This will not only amplify the sensitivities in graphene-based biosensors, but also provides a non-toxic and scalable substitute to typical hazardous wet chemical methods, such as hydrazine or sodium borohydride treatments, which may introduce significant impurities and defects.

Moreover, plasma-treatment of graphene promotes the tailoring of functionalities for the realization of specific biorecognition domains. In particular, the controlled doping and oxidation of graphene outlines the two essential plasma-specific activations. Firstly, N_2_ plasma may be utilized to simultaneously dope and reduce GO to a high quality [136] (Figure 2e). Indeed, the doping of graphene enables the attachment of amino functional groups which facilitate the immobilisation of analyte-specific bioreceptors (e.g., antibodies, antigens, or ssDNAs) to enable specificity in biosensing. Moreover, as the photoluminescence of graphene may be enhanced upon oxidation with O_2_ plasma, this presents an opportunity to fabricate highly-sensitive and selective platforms for FRET-based protein recognition by a faster, cheaper, and health-benign alternative of plasma-activation. This is important because chemically toxic treatments involving concentrated acids may induce significant substrate defects or unwanted impurities. Lastly, the plasma treatment may be utilized to produce GO-NP composites with a good dispersion of NPs (Figure 2f) [137,138].

## 3. Carbon Nanotubes

Carbon nanotubes (CNTs) are hollow graphitic nanomaterials comprised of cylindrical *sp*^2^-hybridized carbon atoms. CNTs exist in two principle forms, single-walled (*i.e.*, SWCNT) and multi-walled (*i.e.*, MWCNT). The former is characterised by a one-dimensional tubular graphene sheet (1–2 nm diameter) [139,140,141], and the latter as concentric, closed graphite tubules, of diameter 2–50 nm with sub-nanometer (~0.35 nm) interlayer spacing [142,143,144]. CNTs have many properties that are desired for biosensors, such as semiconducting characteristics, excellent electrical conductivity, high aspect ratio, mechanical integrity, and chemical stability. Consequently, CNTs have been integrated into numerous electrical (Section 3.1) and optical (Section 3.2) platforms, for the determination of biological complexes including, but not limited to, nucleic acids and protein biomarkers. In addition, due to the unique morphology of CNTs, three variants in biorecognition substrates, *namely*, single CNT platforms, randomly distributed CNT arrays, and vertically aligned CNT forests, have been realized.

### 3.1. CNT-Based Electrical Biosensors

**Figure 3:**
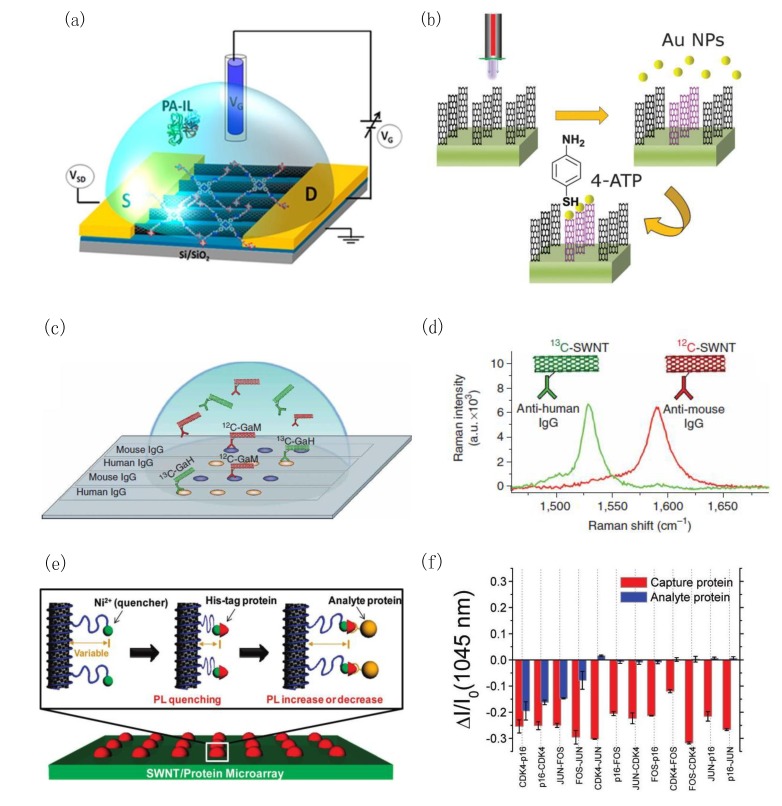
.Carbon nanotubes (CNT)-based biosensors including FETs, vertically aligned CNT arrays for SERS, CNTs for Raman tags, and fluorometric CNT arrays. (**a**) Schematic of an electrolyte-gated CNT-FET, in which CNT walls were noncovalently functionalized with porphyrin-based glycoconjugates. Reproduced from [162]; (**b**) Outline of the vertically aligned CNTs functionalization with AuNDs with an atmospheric pressured plasma jet (APPJ). Reproduced from [163]; (**c**) Multi-color SWCNT Raman labels for multiplexed protein detection; (**d**) Demonstration of the G-mode Raman scattering spectra of ^12^C (red) and ^13^C (green) SWCNT Raman tags. Reproduced from [164]; (**e**) A SWCNT/chitosan suspension was spotted on glass and functionalized with Ni-NTA to bind His-tag-containing capture proteins; (**f**) Illustration of the fluorescence change (Δ*I/I*_0_) following the immobilization of capture proteins and its subsequent binding with the analyte proteins on the SWCNT/chitosan array. Reproduced from [165].

The ability of CNTs to promote charge transfer has been a crucial aspect to its multiple successes in implementations towards electrical biosensors. CNT-based substrates with FET characteristics have been extensively investigated and tailored for protein sensing and DNA sequencing [145,146,147,148,149,150,151,152,153,154,155,156,157], as they offer a highly-sensitive, label-free, and real-time detection. Typically, target analytes are detected by measuring a change in electrical conductivity across the CNT-FET channel caused by their binding with bioreceptors immobilized on the CNT surface. In FETs with horizontally aligned arrays of CNTs (Figure 3a), the detection of long or large biological complexes (e.g., DNAs, proteins) is challenged by a significant Debye screening beyond the CNT double layer. The strategies to overcome this problem have involved the use of small (few nm) receptors including, aptamers [151,158,159,160] and antibody-binding fragments [157,161], functionalized via NPs or chemical linkers with space-modifiers, on the walls of CNTs [153]. Yet another approach recently proposed by Oh *et al.* [145], involved the integration of CNTs to metal-semiconductor FETs (MESFETs). In particular, Oh *et al.* have designed a CNT-MESFET for a label-free, rapid, and highly sensitive (1 pg/mL) determination of Aβ in the human serum. A gold top gate was deposited on the middle of the CNT channel, where probe antibodies (protein A) were immobilized its surface. Subsequently, *E. coli* outer membranes were introduced to inhibit the non-specific binding of Aβ. The achieved 1 pg/mL detection limit not only presents a superior sensitivity to ELISAs for plasma Aβ, but also falls within the physiological range of Aβ in human serum.

### 3.2. CNT-Based Optical Biosensors

The electronic and structural properties of SWCNTs have been essential towards its successful integration in optical biosensors. In particular, the (i) excellent Raman scattering cross-section of SWCNTs, and (ii) its ability to enhance Raman signals near optical transition [166], in conjunction with (iii) its near-infrared (NIR) band-gap fluorescence (*i.e.*, semiconducting SWCNTs) [167], have been exploited in the design of highly-sensitive and selective optical assays for protein biomarkers and DNA sequences. On one hand, this includes SERS-active [163,168,169,170] label-free platforms, and on the other, fluorometric assays [164,165,171,172] and CNT Raman labels [164] in multiplexed biorecognition assays. Notably, Yick *et al.* [163] have demonstrated a hybrid three-dimensional (3D) nanostructure of vertically aligned carbon nanotubes (VACNTs) for an effective microscopic area-selective sensing platform based on SERS (Figure 3b). An atmospheric-pressure microplasma jet (APPJ) was utilized to produce 3D microfluidic channels on the dense arrays of multi-walled VACNTs. Correspondingly, these plasma-treated (hydrophilic) channels were chemically functionalized with Au nanodots, through their ability to confine the Au nanodot aqueous solution. Moreover, these NDs may be adapted to function as preferential sites for the subsequent attachment and sensing of biomolecules (e.g., antibodies, antigens, DNAs, *etc.*) onto these hybrid structures limited to the plasma-modified VACNTs. Chen *et al.* [164] have realized a novel multiplexed protein detection array, in which macromolecular SWCNTs were functionalized as multicolor Raman labels. In particular, Raman detection benefits from the sharp scattering peaks of SWCNTs with minimal background interference, and thus provides a high signal-to-noise ratio for ultra-sensitive detection. In combination with SERS-active substrates, the strong Raman intensity of SWCNT tags enabled protein detection sensitivity to 1 fM. In this assay, SWNT Raman tags were utilized to detect human autoantibodies against proteinase 3, a biomarker for the autoimmune Wegener’s granulomatosis disease. Various antibodies were conjugated to ^12^C and ^13^C SWCNT isotopes (Figure 3c,d), and a multiplexed two-color SWCNT Raman-based protein assay was established. In addition, Ahn *et al.* [165] have harnessed the fluorescence of SWCNTs to pioneer the first chemical approach for a selective and label-free microarray for single protein recognition (Figure 3e,f). In particular, hexahistidine-tagged capture proteins directly expressed by cell-free synthesis on a SWCNT/chitosan microarray were bound to a Ni^2+^ chelated by Nα,Nα-bis(carboxymethyl)-L-lysine grafted to chitosan surrounding the SWCNT. The Ni^2+^ functioned as a proximity quencher with the Ni^2+^/SWCNT distance altered upon docking of the analyte proteins. This ability to discern single protein binding events decreased the detection limit from 100 nM, for the ensemble average, to 10 pM for an observation time of 600 s. More importantly, the versatility of cell-free synthesis to functionalize a nanosensor may be easily extended to include AD or PD biomarkers as capture proteins.

### 3.3. Plasma-Processing: CNTs

**Figure 4:**
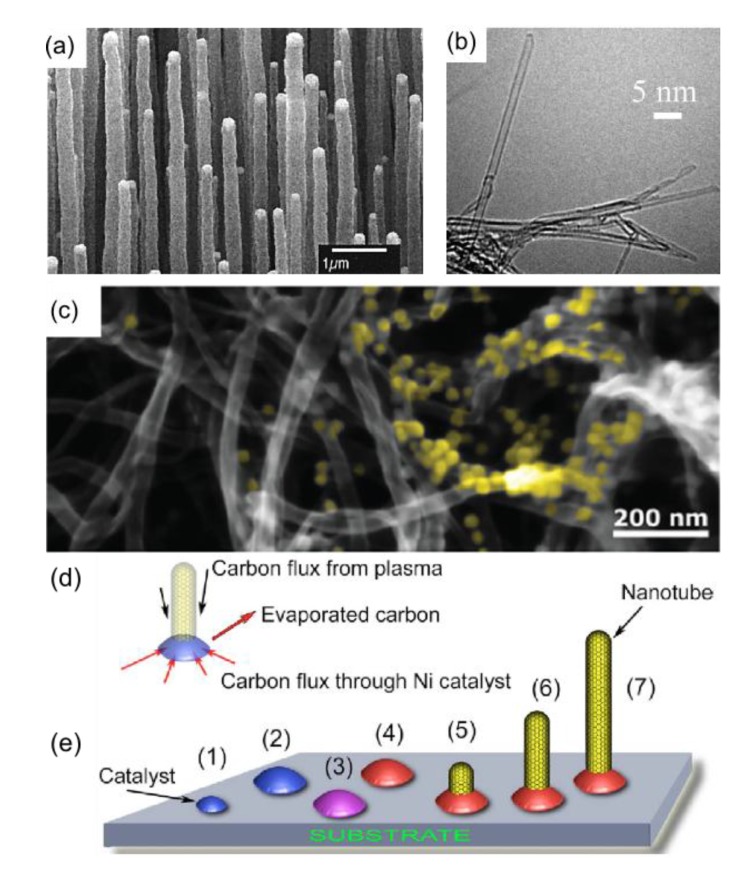
Plasma-enabled growth of vertically aligned carbon nanotubes (CNTs), including CNT structures, morphologies, AuNP modification and growth mechanism. (**a**) MWCNTs fabricated on nickel-coated glass by PECVD. Reproduced from [185]; (**b**) TEM of SWCNTs deposited on SiO_2_ wafer/Si substrate via PECVD (<750 °C) with fast heating. Reproduced from [186]; (**c**) Vertically aligned CNTs treated by cold atmospheric-pressure micro-plasma jet (APMPJ) for the decoration of AuNDs. Reproduced from [163]; (**d**) Overview of CNT growth from CNP nucleation to termination; (**e**) The seven main stages of the formation of the carbon nanostructure pattern on the surface. Reproduced from [30].

Plasma-processing of CNTs provides a practical and efficient tool for the independent control of surface temperature and surface passivation towards a deterministic control over nucleation, self-organisation and functionalization of the arrays of surface-supported CNTs [173,174]. Indeed, plasma-enabled CNT fabrication features remarkably lower temperatures (450 °C) [175,176,177], faster growth rates [178,179], denser growth patterns [180], and excellent morphological and structural features including superior defect-free SWCNTs, and a uniform distribution of CNT lengths and thickness across large patterns and arrays [180,181], as opposed to the conventional methods based on thermal CVD. The structures, morphologies, and the corresponding plasma-enabled growth mechanism for CNTs [180,182] are outlined in Figure 4. Figure 4a exemplifies a highly uniform and dense array of MWCNTs, grown by PECVD on nickel-coated glass. Acetylene gas was used as the carbon source, with ammonia gas for catalysis and dilution. Correspondingly, nanotubes with controllable diameters from 20 to 400 nanometers and lengths from 0.1 to 50 micrometers were obtained, with densities of 10^7^ CNTs/mm^2^. In addition, these unique plasma-based treatments may be tailored to control the morphology of SWCNTs. In particular, Figure 4b illustrates a TEM of SWCNTs deposited on SiO_2_ wafer/ Si substrate via PECVD (<750 °C) with fast heating, for the pyrolysis of pure C_2_H_2_ carbon source at low pressure (30 mTorr), in order to obtain a high yield of semiconducting SWCNTs.

Moreover, the incorporation of metal catalyst nanoparticles (CNPs), while essential for nucleation in the conventional thermal CVD synthesis of CNTs, remains detrimental for two primary reasons. Firstly, they impede direct integration into Si-dominated nanodevice platforms; and secondly, they contribute towards Ohmic losses in charge carrier transport along CNTs. Indeed, plasma processing in CNT growth addresses this concern at two fronts. Firstly, the direct integration of CNTs onto a Si nanodevice was enabled by high-density plasma treatment (CH_4_/N_2_ PECVD at 800 °C, high-vacuum 10^−4^ Pa) [183]. Secondly, plasma-enabled CNT growth demonstrates a catalyst-free synthesis of linear arrays of vertically aligned CNTs (VACNTs), with variable and graded morphologies and structures tailored by the plasma density [183,184].

Lastly, Figure 4d,e outline the growth of CNTs from CNP nucleation to termination, and the balance of carbon fluxes on surface-bound catalyst NPs/carbon nanostructures. In Figure 4e, the seven main stages of the formation of the carbon nanostructure patterns on the surface are highlighted. Sequentially, this involves the (1) nucleation of metal CNPs on the surface; (2) growth of metal CNPs on the substrate surface; (3) carbon supply to the surface, formation of carbon fluxes on the substrate surface between the CNPs, initial saturation of metal CNPs with carbon; (4) formation of carbon-saturated metal NPs; (5) nucleation of carbon nanostructures on the carbon saturated metal catalyst particles; (6) growth of nanostructures on the carbon-saturated metal CNPs due to carbon influx through Ni; and (7) vertical growth due to carbon influx through Ni and carbon removal due to sputtering.

In general, plasmas can modify different nanostructures in a relatively homogeneous way and the stability of modified nanostructures can fulfill the real applications, provided that suitable plasma conditions are implemented. For example, the fabrication of an SERS-based protein sensing platform, *namely*, a dense structure of vertically aligned CNTs and 4-ATP functionalised AuNDs, was enabled by a rapid (10–30 s) cold atmospheric-pressure micro-plasma jet (APMPJ) treatment [163]. In contrast to the conventional low-pressure plasma systems, APMPJ operates at low (mW-W) power, room temperature and atmospheric pressure. Moreover, plasma-assisted CNT synthesis enables a narrower chirality distribution of SWCNTs, and a pronounced vertical alignment to the substrate. The former features a production of semiconducting SWCNTs in large fractions (96%) [186], as opposed to the conventional thermal CVD [187]. Furthermore, a controlled transition of SWCNTs from metallic to semiconducting via H_2_ plasma treatment [188] facilitates the operation of FET-based CNT protein biosensors. Additionally, the surface of CNTs may be activated with amino and carboxyl functional groups, to enable the immobilisation of probe biomolecules including, but not limited to, antibodies, antigens, and aptamers [160,189]. Notably, a low temperature (NH_3_ [190] or CF_4_ [191]) plasma-assisted activation of CNT surface demonstrates a superior and controlled functionalization, via an energy efficient, inexpensive, and significantly less destructive procedure as opposed to the hazardous chemical doping.

## 4. Carbon Nanowalls

Carbon nanowalls (CNWs), also known as vertically-oriented graphenes (VGs), are self-organised and open 3D inter-networked arrays of graphene nanosheets, with individual nanosheets composed of few-layered graphenes, of subnanometer interlayer spacings (0.34–0.37 nm) spanning several micrometers in lateral dimensions [192,193,194,195]. The unique morphology of CNWs presents a high density of (open) active graphitic edges and free active basal planes standing on a substrate. This is complemented by their exceptional tensile strength, high electrical conductivity, semiconducting nature, chemical stability, and ease of functionality [196].

### 4.1. CNW-Based Biosensors

**Figure 5:**
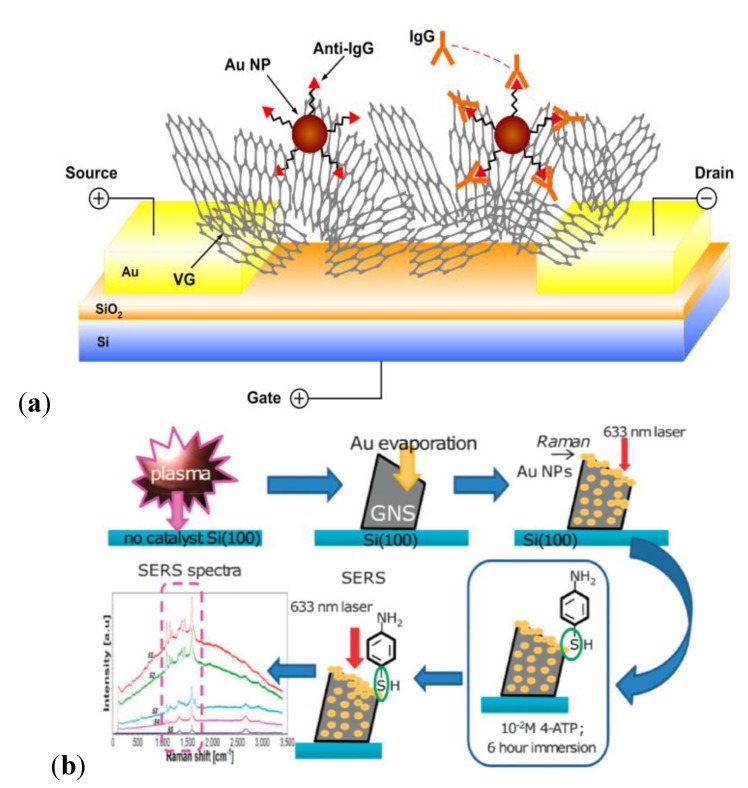
Carbon nanowalls (CNW)-based FET and plasmonic/SERS-active biosensing platforms. (**a**) A CNW substrate was decorated with AuNP-anti-IgG conjugates by electrodeposition, for the sensing of complementary IgGs. Reproduced from [198]; (**b**) CNWs synthesized on Si(100) substrate via a catalyst-free N_2_ PECVD. The thermally evaporated AuNPs were self-assembled onto the CNWs as a SERS platform. Reproduced from [202].

The aforementioned unique features of CNWs have been harnessed in the design of FET devices [197] for immunosensing [198], electrochemical platforms for sequence-specific DNA detection [199,200], and novel absorbance [201] and plasmonic/SERS-active substrates [202,203] which may be tailored for the sensing of AD or PD biomarkers. For instance, Mao *et al.* [198] have developed a sensitive and selective FET biosensor with CNWs labeled with AuNP-antibody conjugates (Figure 5a). CNWs were directly grown on the sensor electrode via a single-step PECVD and functioned as a sensing channel. Subsequently, the protein detection was accomplished through the measurement of changes in the FET channel resistivity upon the antibody-antigen binding. Selectivity against immunoglobulin G (IgG), immunoglobulin M (IgM) and horse radish peroxide (HRP) was demonstrated at the ~2 ng/mL level. Moreover, this presents a single-step and reliable approach which may be adapted to prepare a label-free and real-time biosensing platform for AD or PD protein biomarkers. Arkhavan *et al.* [199] have fabricated a graphene oxide nanowall (GONW) electrode for a label-free and highly-sensitive (0.1 fM resolution) electrochemical detection towards the four bases of dsDNA and the SNPs of oligonucleotides. In particular, GONWs were electrophoretically deposited on a graphite electrode, and the oxidation signals of individual nucleotide bases were monitored by differential pulse voltammetry (DPV). Recently, Seo *et al.* [201] have pioneered (Ar/H_2_) plasma-grown VGs by reforming natural honey precursors, which were subsequently decorated with AuNPs for Au-antibody sensing, whereby a SERS/SPR sensing platform may be facilitated by AuNPs (signal amplification) and shift in UV-Vis diffuse reflectance spectra. In particular, the N_2_ plasma treatment of the VGs enabled the tuning of UV-Vis transmission in VGs for protein sensing (e.g., BSA immobilised on VGs) via Raman spectroscopy and UV-Vis spectrophotometry [202]. Lastly, Rider *et al.* [202] have established a 3D SERS/plasmonic sensing platform based on a plasma-enabled and catalyst-free synthesis of CNWs decorated with self-organized AuNPs (Figure 5b). The controllable high “bookshelf-like” surface coverage of the CNW basal plane by self-organized AuNP arrays was achieved through the use of weakly ionized plasma-based processes. Consequently, the Raman fingerprints corresponding to the radial breathing-modes of CNWs were significantly amplified due to the presence of Au NPs.

### 4.2. Plasma-Processing: CNWs

**Figure 6:**
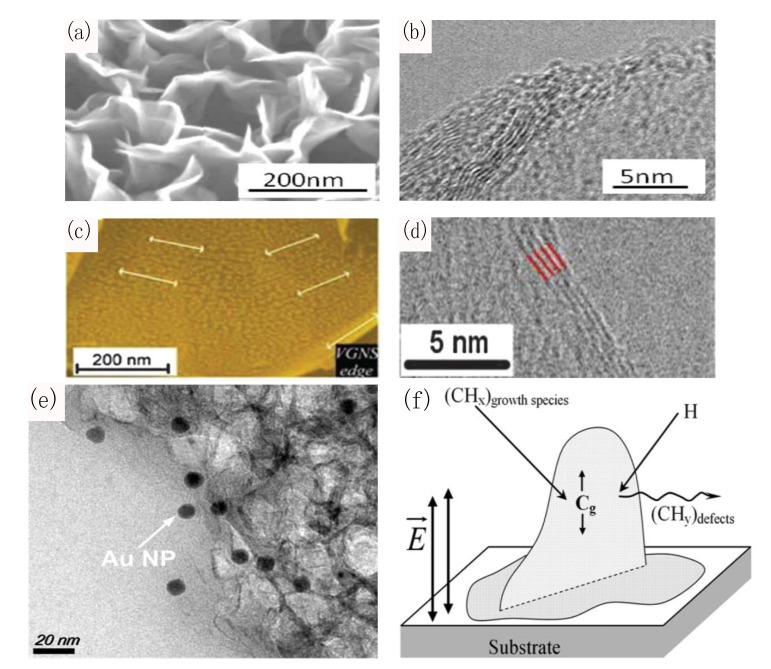
Structures, morphologies, and growth mechanism for plasma-enabled catalyst-free growth of few-layer CNWs. (**a**) High-resolution SEM of ultra-thin CNWs produced from natural honey; (**b**) high-resolution TEM image of the graphitic edges of CNWs. Reproduced from [201]; (**c**) CNWs with self-organised AuNP arrays; (**d**) high-resolution TEM image of 4-layer VGs. Reproduced from [202]; (**e**) AuNPs introduced into CNWs by electrodeposition. Reproduced from [198]; (**f**) growth model for CNWs. Reproduced from [215].

First and foremost, plasma processing of CNWs provides a fast, single-step, energy-efficient and scalable fabrication with superior quality compared to VGs prepared via thermal CVD. In addition, functionalizing CNWs through plasma-activation presents an eco-friendly and inexpensive procedure as opposed to hazardous wet-chemical methods which may introduce significant defects and impurities. Figure 6 illustrates the structures, morphologies and plasma-enabled growth mechanism of CNWs. In particular, CNW fabrication by PECVD via MW (CH_4_/H_2_), RF (CH_4_/Ar), electron beam excited (CH_4_/H_2_), helicon wave-excited (CH_4_), and DC (CH_4_/H_2_/Ar) plasmas [204,205,206,207,208], have demonstrated a catalyst-free, energy-efficient, and low-temperature (400–800 °C) alternative to non plasma-assisted methods including, sputtering (glassy carbon target) in CH_4_/Ar gas [209] and hot filament CVD in a CH_4_/H_2_ or CH_4_/He background gas [210,211,212], which primarily utilize expensive purified hydrocarbon gases in high-temperature environments (700–1000 °C) while involving long fabrication times (20–45 min). Furthermore, recent advances in CNW synthesis has established a single-step, fast, scalable and environmentally-benign (Ar/H_2_ RF) plasma-enabled CNW growth from natural precursors, demonstrating good VG-substrate adhesion and controllability over the degree of graphitization and density of VG edge planes [213,214].

## 5. Conclusions and Outlook

The excellent inherent electrical, optical, and structural properties of CNs render them essential for implementation in biosensors and bioassays. In addition, the sensing of biomarkers indicative of presymptomatic NDs (e.g., AD or PD) requires a biorecognition platform with unprecedented specificity and sensitivity in complex biological fluids such as blood and CSF directly. Moreover, a reproducible, inexpensive and resource-efficient device fabrication is necessary in order to develop a practical biosensor. To this end, we suggest an electrochemical CNW-based substrate to be the most viable platform for the detection of AD or PD biomarkers. CNWs can be directly grown on various conductive substrates so as to reduce the fabrication steps and cost associated with the production of the sensing systems. At present, while only a handful of CNWs-based biosensors for metabolites, proteins and DNAs have been developed, they have consistently demonstrated comparable or improved sensitivities, selectivities, dynamical ranges, and fabrication costs, as compared to biosensors made of CNTs and graphene (see Table 1). In particular, the ease of electrode fabrication, coupled with the inherent properties of graphene, reactive open edges and high NP loading capacity of CNWs, may facilitate a highly sensitive, selective and rapid biosensing device. Consequently, an electrochemical sensor based on CNWs decorated with probe-functionalized (e.g., complementary antibody, fragments, or ssDNA) NPs may provide the most effective approach for a selective determination of CSF protein biomarkers, and a sequence-specific detection of DNA biomarkers. In addition, probe biomolecules may be customized to capture the neurotoxic Aβ42 oligomeric species while in human CSF [216], target specific regions of Aβ for the differentiation between various oligomers [29] and conformers [217,218], or be replaced with phospholipid-specific biomolecules [219], for the detection of blood-based phospholipid biomarkers. Alternatively, Aβ-specific ligands may be employed as bioreceptors ([220] and references therein) to reduce the fabrication costs associated with antibody or antigen probes.

Moreover, plasma-based techniques for fabrication and surface treatment may be implemented to most effectively enable, enhance, and tailor the device performance and optimize the fabrication processes of CNs towards the construction of biosensing devices with superior properties, via a plethora of energy-efficient, environmentally-benign, and inexpensive approaches, for future healthcare applications. In particular, by harnessing these unique and beneficial plasma-based techniques for the surface modification of CNWs, this highlights a great promise towards the fabrication of an ultrasensitive, ultraselective, and cost-efficient biosensor, for an early diagnosis of NDs. More specifically, these plasma-based approaches offer a highly effective and efficient functionalization of CNs. Consequently, this promotes a facile immobilization of highly specific probe biomolecules (e.g., antibodies, antigens, aptamers, and other selective receptors), and hence, may enable a highly versatile and selective biorecognition device capable of detecting ND biomarkers in complex biological fluids such as blood and CSF. Furthermore, these sensing strategies may be tailored to facilitate a range of highly promising therapeutics for the prevention and treatment of NDs. For example, this may involve the inhibition of biomolecules such as mRNA [221], β secretase [222], and interleukin-12/23 [223], whose expressions are crucial in the early stages of ND pathogenesis. However, further research in the biocompatibility of CNs remains highly warranted [224]. Notably, the integration of plasma-enabled CNs in the next-generation of biosensors presents a promising solution for the realization of devices, which may be tailored for the early diagnosis of a diverse spectrum of life-threatening diseases (e.g., diabetes, cancer, and cardiovascular diseases) in addition to the neurodegenerative diseases.

**Table 1:** A survey of CN-based biosensors that have been realized for the detection of metabolism-related biomolecules, DNAs and proteins. These biosensors have been selected to illustrate the varying specificity, sensitivity and fabrication approaches for various CN substrates (*i.e.*, CNWs, CNTs or graphene), biomolecules of interest, and transduction mechanisms.

Sensors	Target analyte	Detection limit	Detection range	Reference
CNWs on graphite electrode	DNAs	9.4 zM	0.1 fM–10 mM	[199]
GO with sulfonated polyalinine	DNAs	5.2 fM	0.1 µM–10 fM	[225]
CNWs on Si	Metabolites	0.17 µM	1–100 µM	[226]
CNWs on Ti-coated Si	Metabolites	0.3 µM	0.01–50 mM	[227]
CNWs on Au	Immunoglobulins	2 ng/mL	2–20 ng/mL	[198]
CNTs	DNAs	100 aM	100 aM–1 pM	[228]
CNT-based FETs	DNAs	0.1 mg/mL	–	[229]
Nitrogen-doped MWCNTs	Glucose	10 µM	0.02–1.02 mM	[230]
MWCNTs with GOx and Au NPs	Glucose	20 µM	0.05–22 mM	[231]
SWCNTs on SiO_2_	Immunoglobulins	1 pg/mL	100 fg/mL–1000 pg/mL	[157]
SWCNTs on SiO_2_	Immunoglobulins	1 pg/mL	1–1000 pg/mL	[145]
Functionalized SWCNTS	Immunoglobulins	1 fM	100 pM–1 fM	[164]
Graphene	DNAs	0.12 pM	1 pM–0.1 µM	[232]
Graphene on SiO_2_	DNAs	10 pM	10 pM–100 nM	[93]
N-doped graphene oxide	Immunoglobulins	0.012 U/mL	0.1–20 U/mL	[75]
Graphene oxide with Au NPs	Immunoglobulins	1 pM	1 pM–1 µM	[70]
Graphene oxide with Au NPs	Immunoglobulins	0.0375 µg/mL	0.0375–40 µg/mL	[110]
